# mTORC1 Signaling in Individual Human Muscle Fibers Following Resistance Exercise in Combination With Intake of Essential Amino Acids

**DOI:** 10.3389/fnut.2019.00096

**Published:** 2019-06-25

**Authors:** Sebastian Edman, Karin Söderlund, Marcus Moberg, William Apró, Eva Blomstrand

**Affiliations:** ^1^The Åstrand Laboratory, the Swedish School of Sport and Health Sciences, Stockholm, Sweden; ^2^Department of Physiology and Pharmacology, Karolinska Institutet, Stockholm, Sweden

**Keywords:** muscle fiber type, protein expression, S6K1, single muscle fiber, EAA

## Abstract

Human muscles contain a mixture of type I and type II fibers with different contractile and metabolic properties. Little is presently known about the effect of anabolic stimuli, in particular nutrition, on the molecular responses of these different fiber types. Here, we examine the effect of resistance exercise in combination with intake of essential amino acids (EAA) on mTORC1 signaling in individual type I and type II human muscle fibers. Five strength-trained men performed two sessions of heavy leg press exercise. During exercise and recovery, the subjects ingested an aqueous solution of EAA (290 mg/kg) or flavored water (placebo). Muscle biopsies were taken from the vastus lateralis before and 90 min after exercise. The biopsies were freeze-dried and single fibers dissected out and weighed (range 0.95–8.1 μg). The fibers were homogenized individually and identified as type I or II by incubation with antibodies against the different isoforms of myosin. They were also analyzed for both the levels of protein as well as phosphorylation of proteins in the mTORC1 pathway using Western blotting. The levels of the S6K1 and eEF2 proteins were ~50% higher in type II than in type I fibers (*P* < 0.05), but no difference was found between fiber types with respect to the level of mTOR protein. Resistance exercise led to non-significant increases (2–3-fold) in mTOR and S6K1 phosphorylation as well as a 50% decrease (*P* < 0.05) in eEF2 phosphorylation in both fiber types. Intake of EAA caused a 2 and 6-fold higher (*P* < 0.05) elevation of mTOR and S6K1 phosphorylation, respectively, in both type I and type II fibers compared to placebo, with no effect on phosphorylation of eEF2. In conclusion, protein levels of S6K1 and eEF2 were significantly higher in type II than type I fibers suggesting higher capacity of the mTOR pathway in type II fibers. Ingestion of EAA enhanced the effect of resistance exercise on phosphorylation of mTOR and S6K1 in both fiber types, but with considerable variation between single fibers of both types.

## Introduction

Human skeletal muscle contains a mixture of fibers with different contractile and metabolic properties, these fibers also differ with respect to adaptability to training. Slow twitch type I fibers have, in general, more mitochondria and a higher oxidative capacity (1, 2). In contrast, type II fibers are more able to produce energy rapidly due to their elevated intracellular level of phosphocreatine and higher glycolytic capacity (3–5).

Resistance exercise enhances the size of individual muscle fibers, in particular fast-twitch fibers (6, 7). Another strong activator of muscle protein synthesis is dietary intake of protein or amino acids (8, 9). The anabolic response evoked by both of these factors is mediated through changes in phosphorylation of proteins in the mechanistic target of rapamycin complex 1 (mTORC1) pathway, including p70 ribosomal protein S6 kinase 1 (S6K1) and eukaryotic elongation factor 2 (eEF2) (10, 11). Furthermore, the extent of phosphorylation levels of these enzymes is an indicator of skeletal muscle hypertrophy and strength adaptation (12, 13). Resistance exercise and intake of protein or amino acids also act synergistically, with the latter potentiating stimulation of mTORC1 signaling (14, 15), as well as protein synthesis by resistance exercise (16).

In an earlier study, we demonstrated that maximal eccentric contractions evoked dissimilar responses in type I and type II fibers: phosphorylation of S6K1 and of ribosomal protein (rp) S6 was elevated 3–4-fold and 6–9-fold, respectively, in type II fibers, with no change in the type I fibers (17). In this study, protein phosphorylation was measured in pools (100–600 fibers) of type I and type II fibers (17). Furthermore, when subjects are provided nutritional supplement shortly before and after resistance exercise, the responses of type I and II fibers appear to be similar (18). Koopman and colleagues, who employed immunohistochemical evaluation of cross-sections of muscle fibers, detected similar elevations in rpS6 phosphorylation in type I and type II fibers during the recovery phase following resistance exercise when protein was administered together with carbohydrates. However, the variation within the different fiber types was not taken into consideration in these studies.

Accordingly, in the present study, the subjects performed a session of heavy resistance exercise in combination with intake of essential amino acids (EAA) or placebo. Muscle biopsies were taken before and after exercise for analysis of individual type I and type II fibers with respect to both the levels of protein and the degrees of phosphorylation of proteins of the mTORC1 pathway. For this purpose, we employed a novel application of the Western blotting technique, an approach similar to that described by Murphy (19). This procedure provides us with a unique insight into muscle adaptation, as well as variations in intracellular signaling within the same type of fiber. We hypothesize that both exercise and intake of EAA will elicit a larger response in the type II than in type I fibers.

## Materials and Methods

### Subjects

Five healthy men who had been performing resistance training for at least 1 year gave both their oral and written consent to participate after being fully informed about the procedure and possible risks involved. Their mean age was 25 ± 3 years, height 179 ± 4 cm, weight 85 ± 4 kg, and maximal one repetition leg press (1RM) 442 ± 18 kg. These subjects were a sub-group from our earlier study (20). The present investigation was approved by the Regional Ethical Review Board in Stockholm.

### Experimental Trial

Following a 9 h fast, subjects reported to the laboratory in the morning. A baseline biopsy was taken from the vastus lateralis muscle with a Weil-Bakesley conchotome (AB Wisex, Mölndal, Sweden) (21) under local anesthesia (Carbocain® 20 mg/ml AstraZeneca AB, Södertälje, Sweden). Thereafter, the subjects performed 10 sets of 8–12 repetitions starting at a load of 85% of 1RM and gradually decreasing with 3 min rests between sets. Muscle biopsies were taken repeatedly during recovery (20), although in the present study only the biopsy taken 90 min after exercise was analyzed since the response was most pronounced at this time point (20). All biopsies were rapidly freed from blood and frozen in liquid nitrogen for storage at −80°C.

At nine time points during the experiment (immediately before and after the warm-up exercise, after the fourth and eighth sets, and following 15, 30, 60, 90, and 120 min of recovery), the participants consumed 150 ml of either an aqueous solution of EAA or placebo (flavored water) in a double-blind, counterbalanced order (20). The EAA mixture consisted of 17.8% L-lysine, 17.1% L-leucine, 14.3% L-phenylalanine, 13.6% L-histidine, 13.6% L-threonine, 11.4% L-valine, 9.5% L-isoleucine, and 2.9% L-methionine (Ajinomoto, Kanagawa, Japan). The total amount of EAA supplied to each subject was 290 mg/kg body weight. Both solutions contained salts and artificial sweetener.

### Tissue Processing

The biopsies were freeze-dried and single fibers dissected out under a light microscope (Nikon, Japan) and each fiber weighed using a quartz-fiber fish pole balance, spectrophotometrically calibrated with p-nitrophenol (22). Fibers weighing < 0.95 μg were found to produce Western blots of unreliable quality and were therefore discarded. A total of 684 muscle fibers were analyzed. The number of fibers analyzed for each subject is presented in Table 1 and the average fiber weight is presented in Table 2.

**Table 1:** Number of fibers analyzed in biopsies from the five subjects (S).

**Subject**	**Type I**				**Type II**			
	**Placebo Pre**	**Placebo 90**	**EAA Pre**	**EAA 90**	**Placebo Pre**	**Placebo 90**	**EAA Pre**	**EAA 90**
S 1	9	10	7	4	12	10	9–10	14
S 2	10–11	9	13	7–9	7	12	9	12
S 3	13–22	4–11	9–15	5–12	9–21	29–30	20	14–28
S 4	9–18	12–15	4–9	3–6	29–38	29–38	36–52	36–49
S 5	11	9–12	8	11–12	26	30	29–30	25–26

*A different number of fibers was in some cases analyzed for the three proteins (indicated by range)*.

*Pre indicates before exercise and 90 indicates 90 min after exercise*.

**Table 2:** Weight of the fibers (μg) analyzed in the different conditions.

	**Type I**				**Type II**			
	**Placebo Pre**	**Placebo 90**	**EAA Pre**	**EAA 90**	**Placebo Pre**	**Placebo 90**	**EAA Pre**	**EAA 90**
mTOR	2.54 (0.95–5.73)	2.38 (1.09–8.10)	2.18 (0.95–4.37)	2.18 (1.09–6.00)	3.03 (1.09–6.00)	2.86 (1.09–7.09)	2.56 (1.23–5.87)	2.46 (1.09–5.73)
S6K1	2.55 (0.95–5.73)	2.26 (1.09–8.10)	2.12 (0.95–4.23)	2.26 (1.09–6.00)	2.99 (1.09–6.00)	2.82 (1.09–7.09)	2.52 (1.23–5.87)	2.32 (1.09–5.46)
eEF2	2.54 (0.95–5.73)	2.43 (1.09–8.10)	2.13 (0.95–4.37)	2.18 (1.09–6.00)	3.03 (1.09–6.00)	2.85 (1.09–7.09)	2.53 (1.23–5.87)	2.37 (1.09–5.73)

*The values are presented as means (range)*.

*Pre indicates before exercise and 90 indicates 90 min after exercise*.

### Homogenization

Each muscle fiber was dissolved in 5 μl homogenization solution consisting of 2 mM Hepes, 1 mM EDTA, 5 mM EGTA, 10 mM MgCl_2_, 1% Triton X-100, 50 mM β-glycero-P, 1 mM sodium ortovanadate, 1% Phosphatase Inhibitor Cocktail 3 (Sigma-Aldrich), 1% Halt™ Protease Inhibitor Cocktail (Thermo Scientific), and 2 mM 4,4′-DDT at pH 7.4. The samples were stored on ice for approximately 1 h throughout homogenization followed by addition of 5 μl 2X Laemmli sample buffer (Bio Rad). The samples were then heated at 95°C for 5 min and stored at −20°C. The procedure is modified from the method originally described by Murphy (19).

### Immunoblotting

The entire 10 μl containing each dissolved muscle fiber was loaded onto a 26 well Criterion TGX gradient gel (4–20% acrylamide; Bio Rad). A supernatant from a whole muscle homogenate of a post-exercise muscle biopsy was also loaded onto each gel to control for between membrane differences in protein phosphorylation and expression. The Western blotting protocol employed has been described in detail previously (20).

In brief, electrophoresis was run for 30 min at 300 V in Tris buffer (25 mM Tris base, 192 mM glycine, and 3.5 mM SDS) on an ice bed in a cold room at 4°C. The gels were subsequently incubated in transfer buffer (25 mM Tris base and 192 mM glycine in 90% dH_2_O−10% methanol) for 30 min at 4°C and the proteins transferred to polyvinylidine fluoride membranes (Biorad) at 300 mA for 180 min at 4°C. The membranes were subsequently stained using Pierce® Reversible Stain Kit (Thermo Scientific).

Prior to incubation with primary antibodies, the membranes were blocked for 1 h at room temperature using 5% milk in Tris buffer-saline (TBS) (20 mM Tris base and 137 mM NaCl). Incubation with primary antibodies targeting the phosphorylated proteins was carried out over-night at 4°C, the membranes were then washed prior to incubation with secondary antibodies for 1 h at room temperature, and then washed once again prior to visualization with SuperSignal® West Femto Maximum Sensitivity Substrate (Thermo Scientific). The Molecular Imager ChemiDoc™ XRS system was used to detect protein bands and the Quantity One® software, version 4.6.3 (Bio-Rad Laboratories) for quantification.

The membranes were subsequently washed thoroughly with dH_2_O (5 ×1 min) and TBS (3 ×3 min) and thereafter stripped for 30 min at 50°C utilizing Restore™ PLUS Western Blot Stripping Buffer (Thermo Scientific). After being washed once again with dH_2_O (5 ×1 min) and TBS (3 ×3 min), the membranes were incubated with primary antibodies for detection of total protein as described above. The levels of phosphorylated and total protein are expressed in relation to the weight of the fiber.

### Fiber Typing

The membranes were again stripped twice prior to exposure of antibodies targeting myosin heavy chain I (MHC I) and then myosin heavy chain II (MHC II). On the basis of visual inspection of the MHC I and MHC II staining, fibers were categorized either as type I, type II, or hybrid fibers (staining for both MHC I and II). The hybrid fibers were discarded; only 22 fibers (about 3% of the fibers) were classified as hybrid fibers, which is too small to give a valuable result.

### Antibodies

Both primary and secondary antibodies were diluted with TBS containing 2.5% milk. The primary antibodies used were Mouse Anti-Slow Skeletal Myosin Heavy Chain (Abcam #ab11083, diluted 1:10,000), Rabbit Anti-Fast Myosin Skeletal Heavy Chain (Abcam #ab91506, diluted 1:10,000), total mTOR (Cell Signaling #2983S, diluted 1:1,000), phosphorylated mTOR Ser^2448^ (Cell Signaling #5536S, diluted 1:1,000), total S6K1 (Cell Signaling #2708S, diluted 1:1,000), phosphorylated S6K1 Thr^389^ (Cell Signaling #9234S, diluted 1:1,000), total eEF2 (Cell Signaling #2332S, diluted 1:1,000), and phosphorylated eEF2 Thr^56^ (Cell Signaling #2331S, diluted 1:1,000). The secondary antibodies, anti-rabbit HRP-linked (#7074S) and anti-mouse HRP-linked (#7076S), were both purchased from Cell Signaling and diluted 1:10,000.

### Statistical Analysis

A repeated measures three-way ANOVA (supplement, fiber type, and time) was applied to compare changes in mean levels of protein expression and phosphorylation in the type I and type II fibers in the two conditions. When a main or interaction effect was detected, planned comparisons was performed to identify where the differences occurred. The level of statistical significance was set at *P* < 0.05. Statistical analysis was carried out with Statistica software (version 12.0, Statsoft, Tulsa, OK).

## Results

### Protein Levels

**Figure 1:**
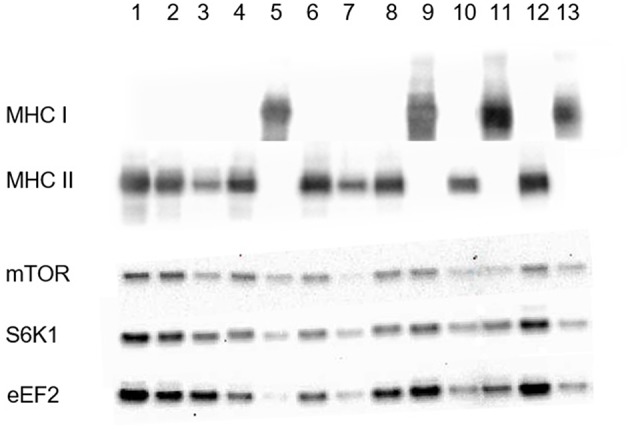
Identification of type I and type II fibers following incubation with antibodies targeting MHCI and MHCII. Lower bands represent protein levels of mTOR, S6K1, and eEF2 in individual fibers. Proteins were separated on 4–20% acrylamide gels and transferred to PVDF membranes.

**Figure 2:**
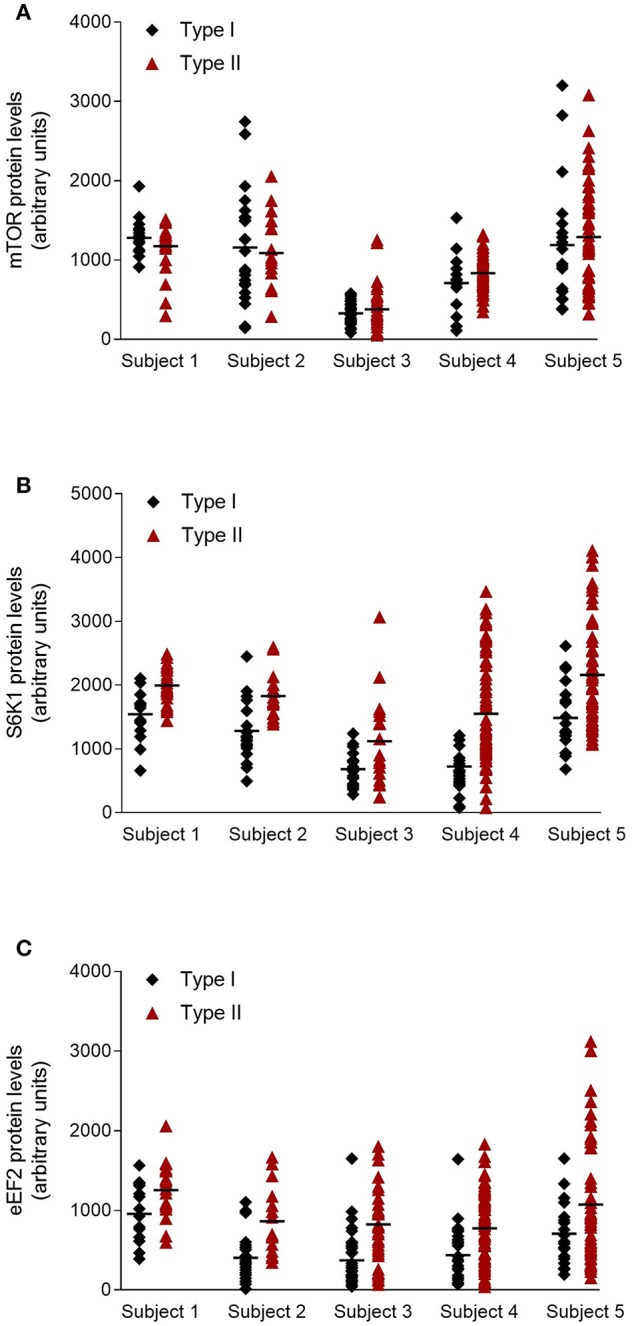
Protein levels in individual type I and type II fibers before exercise from the five subjects. **(A)** mTOR, **(B)** S6K1, and **(C)** eEF2. Horizontal lines illustrate the average values.

Figure 1 shows a representative picture of fiber type identification as well as protein levels of mTOR, S6K1, and eEF2 in type I and type II fibers. The levels of both the S6K1 and eEF2 proteins were significantly higher in type II than type I fibers (50% for both proteins, *P* < 0.05), whereas no difference was observed with respect to the mTOR protein (Table 3). With the exception of a significant increase in the level of mTOR following exercise, no main effect of time or supplementation was observed. The variability in protein levels between single muscle fibers within and between subjects is illustrated in Figure 2.

**Table 3:** Protein levels of mTOR, S6K1 and eEF2 in type I and type II muscle fibers.

		**Type I**		**Type II**	
**Protein**		**Pre**	**90**	**Pre**	**90**
mTOR	Placebo	965 ± 161	1115 ± 164*	962 ± 133	1027 ± 146*
	EAA	868 ± 184	1106 ± 201*	939 ± 159	1177 ± 176*
S6K1	Placebo	1181 ± 174	1055 ± 156	1828 ± 154^*$*^	1669 ± 136^*$*^
	EAA	1084 ± 171	1272 ± 155	1647 ± 173^*$*^	1558 ± 113^*$*^
eEF2	Placebo	564 ± 93	629 ± 99	995 ± 92^*$*^	912 ± 124^*$*^
	EAA	586 ± 131	596 ± 130	926 ± 110^*$*^	818 ± 97^*$*^

The values presented (arbitrary units) are means ± SE for five subjects. *P < 0.05 vs. Pre (main effect of time), and ^$^P < 0.05 vs. type I fibers (main effect of fiber type).

*Pre indicates before exercise and 90 indicates 90 min after exercise*.

### Protein Phosphorylation

**Figure 3:**
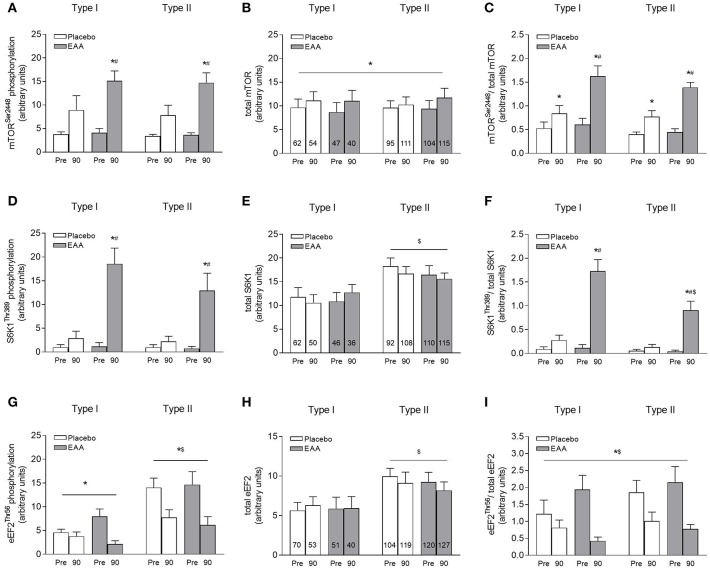
**(A)** Phosphorylation of mTOR at Ser^2448^, **(B)** total protein level of mTOR, **(C)** phosphorylation of mTOR/total protein, **(D)** phosphorylation of S6K1 at Thr^389^, **(E)** total protein level of S6K1, **(F)** phosphorylation of S6K1/total protein, **(G)** phosphorylation of eEF2 at Thr^56^, **(H)** total protein level of eEF2, and **(I)** phosphorylation of eEF2/total protein. Pre indicates before exercise and 90 indicates 90 min after exercise. Figures within bars indicate the number of fibers analyzed. The values presented (arbitrary units/100) are means ± SE for the five subjects. *P < 0.05 vs. Pre, ^#^*P* < 0.05 vs. placebo, and ^$^*P* < 0.05 vs. type I fibers.

A main effect of time was found for phosphorylation of both mTOR^Ser2448^ and S6K1^Thr389^, in addition to an interaction between time and supplement. The planned comparison analysis revealed an increase in both fiber types 90 min after exercise in the EAA condition only, despite a 2–3-fold increase in the levels of phosphorylated mTOR (*P* = 0.085) and S6K1, in the placebo condition (Figures 3A,D). However, when phosphorylation was normalized to the level of the corresponding protein, the increase in mTOR phosphorylation proved to be significant (Figure 3C). There were no differences between fiber types and no interaction involving fiber type with respect to mTOR or S6K1 phosphorylation, other than when the S6K1 phosphorylation was normalized to the total level of corresponding protein. In this case phosphorylation of S6K1 was higher in type I than in type II fibers 90 min after exercise (interaction between fiber type and supplement; *P* < 0.05, Figure 3F).

Phosphorylation of eEF2 at Thr^56^ was associated with a main effect of both time and fiber type. An interaction between time and fiber type was also found, with no main or interaction effects involving supplement. At rest, phosphorylation of eEF2 at Thr^56^ was 128% higher in type II than type I fibers (*P* < 0.05, Figure 3G). Following exercise, the phosphorylation was reduced by 53% in both types of fiber (*P* < 0.05 compared to Pre, Figure 3G). When the level of phosphorylation was normalized to the level of protein, the ANOVA did not reveal a significant interaction between fiber type and time. Hence, an overall higher phosphorylation in type II than in type I fibers, including both resting and exercised levels, was detected (Figure 3I).

**Figure 4:**
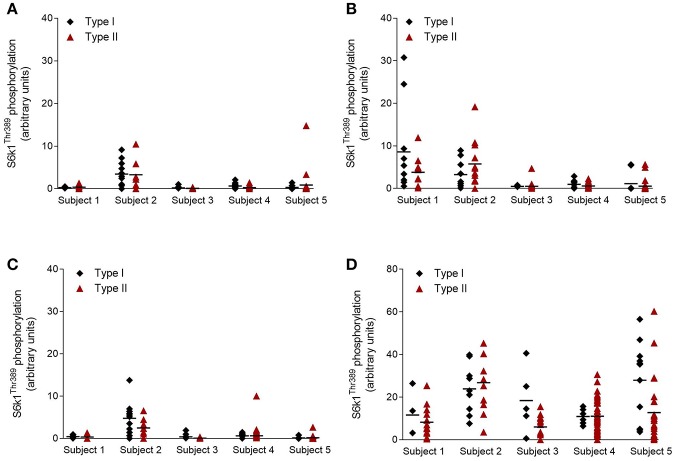
Phosphorylation of S6K1 at Thr^389^ in individual fibers of five subject. **(A,C)** Levels at rest prior to exercise and supplementation. **(B)** Levels following exercise in placebo condition, and **(D)** levels following exercise and supplementation with EAA. Horizontal lines illustrate the average values. Y-axis in **(D)** (0–80) is different from **(A–C)** (0–40).

Figure 4 illustrates the variability in phosphorylation status of S6K1 between individual fibers within and between subjects before and 90 min after exercise in both conditions.

## Discussion

It is well-known that human skeletal muscle contains fiber types with different metabolic properties and the vast majority of studies in this area have examined whole muscle. In the present study, we have isolated and analyzed individual muscle fibers with respect to protein components of the mTORC1 signaling pathway. The major findings are (1) type II fibers express higher levels of the S6K1 and eEF2 proteins than type I fibers, (2) type I and type II fibers respond similarly to intake of EAA in combination with resistance exercise, and (3) there is a large variation in this response between individual fibers within each subject as well as between subjects.

Type II muscle fibers had approximately 50% higher levels of both S6K1 and eEF2, signal transduction proteins downstream of mTOR. These findings are in agreement with a previous report on rat muscle where the levels of S6K1 and protein kinase B (Akt/PKB) are higher in the fast EDL than in the slow soleus muscle (23). These proteins are known to be involved in regulating translation initiation or elongation processes in skeletal muscle (24), but the functional consequences of the elevated protein levels of S6K1 and eEF2 in type II human muscle fibers are not entirely clear. However, it may suggest a larger capacity of the mTORC1 pathway, i.e., a higher activity of key enzymes in the pathway provided that anabolic stimuli induce similar degree of phosphorylation of these enzymes, which may explain why hypertrophy can be stimulated to greater extent in type II fibers (6, 7).

Resistance exercise alone did not increase the phosphorylation of mTOR and S6K1 significantly, although the levels were elevated by 2–3-fold 90 min after exercise. This differs from our previous observation that maximal eccentric contractions markedly elevated phosphorylation of S6K1 and reduced phosphorylation of eEF2 in type II fibers only (17). The failure to detect such a difference here is most probably due to the differences between the exercise protocols utilized. Maximal eccentric contractions impose a pronounced strain on the muscle, the force is ~30% higher than maximal concentric contractions (25) together with the lengthening and stretching of the muscle fiber. The latter phenomenon is an important component of the training response, as well as for activation of S6K1 (26, 27). Despite the heavy work performed by our subjects, the leg press exercise involves both eccentric and concentric movements. The initial exercise at 85% of 1RM, gradually reducing the load to 65% of 1RM, entails a relatively low contribution from the eccentric phase to force development, as well as less strain during the lengthening and stretching of the muscle fibers. In addition, repeated sets of resistance exercise with short rest periods between sets have been suggested to fatigue primarily the type II fibers (28). This may imply that the type of exercise employed here could have fatigued the type II fibers, and hence, explaining the larger involvement of type I fibers as compared to maximal eccentric contractions.

When the subjects ingested a mixture of EAA, mTOR, and S6K1 were stimulated considerably in both type I and type II fibers following resistance exercise (Figure 3). Interestingly, but in contrast to our hypothesis, when normalized to the total level of protein, S6K1 was significantly more phosphorylated in type I than type II fibers, suggesting that amino acid-induced stimulation of translation initiation is more responsive in type I fibers. This finding implies that type I fibers may have a higher growth potential following amino acid ingestion, given the positive associations between S6K1 phosphorylation and muscle hypertrophy (12, 13). However, this notion is difficult to reconcile with previous reports showing that fiber type enlargement following resistance exercise occurs primarily in type II fibers (6, 7). While it is generally accepted that the protein synthetic response is the major determinant of muscle growth (10), it should be emphasized that for muscle accretion to occur, both acutely and in the long term, accumulated increases in muscle protein synthesis must be higher than those of muscle breakdown. Consequently, measurements pertaining only to one side of the protein balance equation may not fully reflect the growth potential of muscle (29). We did not measure fiber type-specific protein synthesis in the present study, but the close agreement generally found between S6K1 phosphorylation and muscle protein synthesis (30–32) suggests that the higher degree of S6K1 phosphorylation found in the type I fibers may be reflective of elevated rates of protein synthesis compared to the type II fibers. This notion is supported by a previous study showing slightly higher rates of muscle protein synthesis in isolated type I compared to type II fibers following resistance exercise in a postprandial state (33).

However, data on resting muscle do not support a larger responsiveness in type I fibers (34, 35). Infusion of a mixture of EAA induced similar increases in the rate of muscle protein synthesis (in both the myofibrillar and sarcoplasmatic fractions) in the soleus, vastus lateralis, and triceps muscles (35). The proportion of type I fibers in these three muscles ranged from 20 to 80% and there was no correlation between the fractional synthetic rate (FSR) and percentage of type I fibers, indicating that type I and type II fibers are equally responsive to hyperaminoacidemia in a resting condition.

Our observations of elevated phosphorylation of eEF2 in type II fibers in resting muscle are in accordance with previous findings (36). Here, the level of eEF2 protein was also higher in type II fibers (Table 3, Figure 3H). The potential consequences of these differences are unclear; it has been suggested that they may reflect a higher resting FSR in type I fibers, which is supported by measurements on pools of fibers revealing a 30% higher rate in type I fibers at rest (37). Further support for this opinion can be found in a study on different human muscles with varying proportions of type I and type II fibers (35), but not in others (34, 38). However, such differences may be more difficult to detect in whole muscle due to the content of both type I and type II fibers as well as hybrid fibers.

We observed here considerable variation in the levels of both protein and phosphorylation between individual fibers of both type I and type II. The large variation in signaling response indicates that some fibers are not recruited, despite the heavy work load. Similar large variations in substrate levels (glycogen) and ATP within fibers of the same type have been reported following endurance exercise or electrical stimulation of the muscle (39, 40), further supporting that only certain fibers are recruited and contribute to force development during muscle contraction. The even larger variation when EAA were ingested is likely caused by the enhancing effect such intake has on fibers that are actually recruited than on inactive fibers (14, 15). The large variation also in protein concentration within the same fiber type (Figure 2) is in line with previous studies showing that both ATP (40) and glycogen (39) vary greatly within fibers of the same type. Although valuable, mean values fail to describe the entire picture within skeletal muscle. Adding single fiber analysis to the otherwise more common whole muscle analysis should add another dimension in understanding the complexity of skeletal muscle adaptation.

In the present study, the number of type II fibers dissected out and analyzed was more than double the number of type I fibers (Table 1). Similar observations have been made previously (5, 41), but we have no explanation to this phenomenon. It could of course reflect the subjects' fiber composition, however, this is unlikely. A more likely explanation is that type II fibers are more robust and easier to dissect without breaking the fibers, meaning that a great amount of fibers need to be dissected out to ensure that a sufficient number of type I fibers is obtained. Dissecting and analyzing single fibers is time consuming, an advantage with the present methodology is that fiber typing and protein analyses are carried out on the same fiber fragment, which shortens the analysis time.

In summary, the levels of both S6K1 and eEF2 proteins were higher in type II than type I muscle fibers, suggesting that in the former the mTORC1 pathway has a greater capacity. Heavy resistance exercise in combination with consumption of EAA elevates phosphorylation of both mTOR and S6K1 in both fiber types above the levels seen at rest and following resistance exercise alone. The only minor differences in the response to intake of EAA was that when normalized to protein content, S6K1 was slightly more phosphorylated in type I fibers. Large variations in the responses of individual fibers to the different stimuli were observed, with some fibers demonstrating high levels of phosphorylation following exercise and others no stimulation at all. Future studies exploring possible relationship between signaling response, on one hand, and substrate levels or metabolic changes, on the other, within individual type I and type II fibers may help to understand the mechanism involved in training adaptation.

## Data Availability

All datasets generated for this study are included in the manuscript and/or the supplementary files.

## Ethics Statement

Five healthy men who had been performing resistance training for at least 1 year gave both their oral and written consent to participate after being fully informed about the procedure and possible risks involved. Their mean age was 25 ± 3 years, height 179 ± 4 cm, weight 85 ± 4 kg, and maximal one repetition leg press (1RM) 442 ± 18 kg. These subjects were a sub-group from our earlier study (20). The present investigation was approved by the Regional Ethical Review Board in Stockholm.

## Author Contributions

SE, KS, and EB designed the study. MM and WA carried out the experiment. SE dissected and analyzed muscle fibers and performed statistical analyses. SE and EB wrote the manuscript. KS, MM, and WA edited the manuscript. All authors approved the final version of the manuscript.

### Conflict of Interest Statement

The authors declare that the research was conducted in the absence of any commercial or financial relationships that could be construed as a potential conflict of interest.
